# Effect of the COVID-19 pandemic on the psychotropic drug consumption

**DOI:** 10.3389/fpsyt.2022.1020023

**Published:** 2022-12-15

**Authors:** Paul Benistand, Philippe Vorilhon, Catherine Laporte, Jean-Baptiste Bouillon-Minois, Georges Brousse, Reza Bagheri, Ukadike Chris Ugbolue, Julien S. Baker, Valentin Flaudias, Aurélien Mulliez, Frédéric Dutheil

**Affiliations:** ^1^Département de Médecine Générale, Université Clermont Auvergne, Clermont–Ferrand, France; ^2^Research Unit AutomédiCation aCcompagnement Pluriprofessionnel PatienT (ACCePPT), Université Clermont Auvergne, Clermont–Ferrand, France; ^3^Clermont Auvergne INP, Centre Hospitalo-Universitaire (CHU) Clermont-Ferrand, Centre National de Recherche Scientifique (CNRS), Institut Pascal, Université Clermont Auvergne, Clermont–Ferrand, France; ^4^Centre National de Recherche Scientifique (CNRS), Laboratoire de Psychologie Sociale et Cognitive (LaPSCo), Physiological and Psychosocial Stress, Centre Hospitalo-Universitaire (CHU) Clermont-Ferrand, WittyFit, Université Clermont Auvergne, Clermont-Ferrand, France; ^5^Department of Exercise Physiology, University of Isfahan, Isfahan, Iran; ^6^School of Health and Life Sciences, University of the West of Scotland, Glasgow, United Kingdom; ^7^Department of Physical Education and Health, Centre for Health and Exercise Science Research, Hong Kong Baptist University, Kowloon, Hong Kong SAR, China; ^8^Laboratoire de Psychologie des Pays de la Loire, Université de Nantes, Nantes, France; ^9^Direction de la Recherche Clinique et Innovations Biostatistics, Centre Hospitalo-Universitaire (CHU) Clermont-Ferrand, Clermont-Ferrand, France

**Keywords:** COVID-19, mental health, psychotropic drug, anxiety, sleep disorder

## Abstract

**Importance:**

Although the COVID-19 pandemic has had a negative impact on mental health, there is no comprehensive longitudinal study of the entire population of a country without selection bias.

**Objective:**

The objective of this study was to evaluate the prescription of psychotropic drugs during the COVID-19 pandemic, using data from the French national health data system (SNDS).

**Design, settings, and participants:**

Prescriptions for psychotropic drugs (antidepressants, anxiolytics, hypnotics, and antipsychotics) from 1 January 2015 to 30 September 2021 were collected from administrative data provided by the SNDS. This database includes more than 99% of the French population, i.e., 67 million people. The data were analyzed using an interrupted time series analysis (ITSA) model.

**Main outcomes and measures:**

Consumption of psychotropic drugs was aggregated in months and expressed in number of boxes per thousand inhabitants.

**Results:**

During the study period, more than 1.3 billion boxes of psychotropic medications were dispensed. Comparison of psychotropic drug dispensing before and after the pandemic showed a relative increase of 0.76 (95 CI 0.57 to 0.95, *p*<0.001) boxes per month per thousand inhabitants, all classes of psychotropic drugs combined. Three classes saw their consumption increase in an almost similar proportion, respectively, by 0.23 (0.15 to 0.32, *p*<0.001) boxes for antidepressants, 0.27 (0.20 to 0.34, *p*<0.001) boxes for anxiolytics and 0.23 (0.17 to 0.30, *p*<0.001) boxes for hypnotics. The change in antipsychotic consumption was very small, with an increase of 0.04 boxes (0.02 to 0.06, *p* = 0.001) per month per thousand population.

**Conclusion and relevance:**

The COVID-19 pandemic had led to an increase in the consumption of psychotropic drugs, confirming the significant impact of the pandemic on the mental health of the general population.

## Introduction

Mental health is a global public health issue (1). The onset of the COVID-19 pandemic in March 2020 has led to major changes in the daily lives of populations with the implementation of government measures to contain the spread of the disease (2). This resulted in the lockdown of billions of people around the world for the first time in human history. Proofs from previous epidemics showed that these isolation measures in an outbreak context were a source of severe mental problems and psychological distress (3, 4). The COVID-19 pandemic, by its magnitude, is no exception, with an increase in depressive symptoms, anxiety, and sleep disorders from the first phase of the pandemic (5–10). However, most studies were cross-sectional, on selected population, and explored mainly the short-term effects of the pandemic. Consumption of anxiolytics, antidepressants, hypnotics, and antipsychotics using administrative data provided by the French National Health System is an alternative method for assessing the mental health of the population (11–13). This method also has the benefits of studying the whole population of an entire country, without any selection bias, using repeated long-term longitudinal data. Considering that several epidemiological studies showed an increase in the consumption of psychotropic drugs following wide-ranging psychological distress such as during natural, (14) industrial, (15) or economic disasters, (16) we hypothesized that the pandemic has led to an increase in the use of medication linked to stress-related disorders.

Therefore, our main objective was to demonstrate the increase in the use of psychotropic drugs following the pandemic, using nation-wide data. More specifically, we intended to describe the delivery of psychotropic drugs in city pharmacies during the 18 months following the onset of the pandemic, in comparison with the previous 5 years.

## Materials and methods

### Database for medication

Data were extracted from National Health Insurance (Sécurité sociale). More precisely, we used the Medic’AM open database, which is part of the SNDS (Système National de Données de Santé) (17). This database is exhaustive, anonymized, and gathers the drugs delivery in city pharmacies–hospital deliveries are not considered. The Medic’AM database, due to its “open status,” only provides aggregated data, with no socio-demographic details. The Medic’AM database collect only the number of boxes delivered per month and per drug classified according to their Anatomical Therapeutic Chemical (ATC) class. Consequently, the Medic’AM database do not have details on the medication packaging (number of pills, dosage, neither posology) preventing us to retrieve the number of defined daily doses (DDD) per 1,000 inhabitants for estimating the medication consumption. Therefore, we decided to consider the number of boxes delivered per habitant as the main indicator of the evolution of psychotropic drugs consumption.

### The Anatomical Therapeutic Chemical classification for medications

The ATC is a pharmaceutical coding system of drugs based on five levels of classification according to the target organ(s) and the therapeutic, pharmacological and chemical properties of each drug (18). The general form of the code for a molecule is LDDLLDD, where L is a letter and D is a digit/number (example: A01AA01). Each letter and each digit doublet represent a successive level. The first level (first letter) defines one of 14 anatomical groups. The second level (first two digits) gives the main pharmacological or therapeutic subgroup. The third and fourth levels (second and third letters) correspond to chemical, pharmacological or therapeutic subgroups. The fifth and last level (last two digits) indicates the chemical substance. The ATC system is controlled by the World Health Organization Collaborating Centre for Drug Statistics Methodology (WHOCC).

### Selected psychotropic medication

In our study, we selected the psychotropic medications based on the fourth level of the ATC. We focused on psychotropic medications including antidepressants (ATC code N06A), anxiolytics (ATC code N05B), hypnotics (ATC code N05C), and antipsychotics (ATC code N05A). We included monthly medication from January 1st, 2015, to September 30th, 2021.

We decided to divide the study period into two time periods. The first, from January 2015 to March 2020, is our control, pre-pandemic period. The second phase, from March 2020 to the end of September 2021, is our COVID-19 pandemic part of the study period.

All persons who had a reimbursed delivery of a psychotropic molecule (ATC code N05A, N05B, N05C, and N06A) in the period from January 2015 to September 2021 were included in this study.

### Statistical analysis

We chose to analyze the data using a long-term Interrupted Series Analysis (ITSA) in order to evaluate the impact of the COVID-19 pandemic on psychotropic’s drug delivery. ITSA has historically been used to evaluate policy impacts and recently often used in studies evaluating COVID-19 pandemic impacts. The main interest of ITSA, is to modelize the pre-pandemic trend and to project it into the pandemic and to use it as counterfactual.

The equation of the model can be written as follows:

$$Y_{t}=\beta_{0}+\beta_{1}\,T_{t}+\beta_{2}\,X_{t}+\beta_{3}\,X_{t}\,T_{t}+\varepsilon_{t}$$

With *Y*_*t*_ the outcome variable measured the month t. *T*_*t*_ is the time since the first measurement (baseline) of the study. *X*_*t*_ is the indicator representing the period (pre-pandemic = 0, pandemic = 1), *X*_*t*_*T*_*t*_ is the interaction term (time x period). β_0_ is the intercept (baseline level of the outcome), β_1_is the slope of the outcome before the pandemic period, β_2_ is the immediate change of the outcome following the start of the pandemic (considered in April 2020), β_3_ is the difference between pre-pandemic and pandemic slopes. This latter parameter represents the main effect tested in our study that should allow to verify if COVID-19 pandemic durably altered psychotropic reimbursement and if so to which extent.

For each psychotropic molecule, crude numbers of boxes reimbursed per months were divided per the total French population according to the INSEE references, in order to adjust medication delivery per month and per habitant. Data was then deseasonalized according to monthly seasonal coefficients (specially for each molecule) calculated from 2010 to 2019. Then analyses were performed–for each molecule of interest- using a long-term Interrupted Series Analysis (ITSA) model using the Stata command ITSA (19) with the deseasonalized number of boxes per 1,000 inhabitants as the outcome (20–22). The data before the 1st lockdown (from 2015 to March 2020) was used to modelized the counterfactual evolution after in order to test changes in trends of medication delivery. Trends coefficient before and after the 1st lockdown, and coefficients of change after relative to before the pandemic are expressed with their 95% confidence interval and plotted accordingly. All tests were two-sided and a *P*-value < 5% was considered significant. Statistics were computed using Stata 15 (StataCorp, College Station, TX, USA).

## Results

### Participants

**FIGURE 1:**
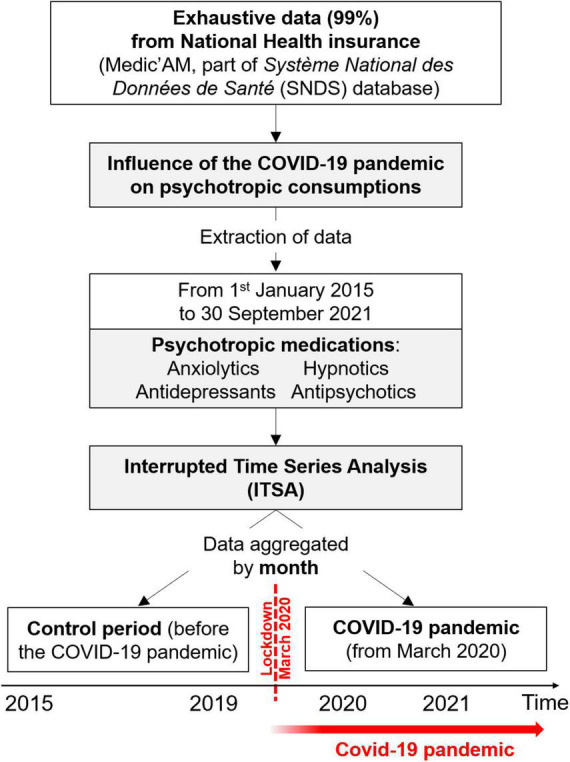
Flow chart.

The database includes more than 99% of the French population, i.e., 67 million people. All persons covered by the health insurance system were included. Since all patients are extracted from a national database, there is no study selection bias or attrition bias. Over the study period, more than 1.3 billion boxes of psychotropic drug were delivered (Figure 1).

### The influence of the pandemic on overall psychotropic medication

**FIGURE 2:**
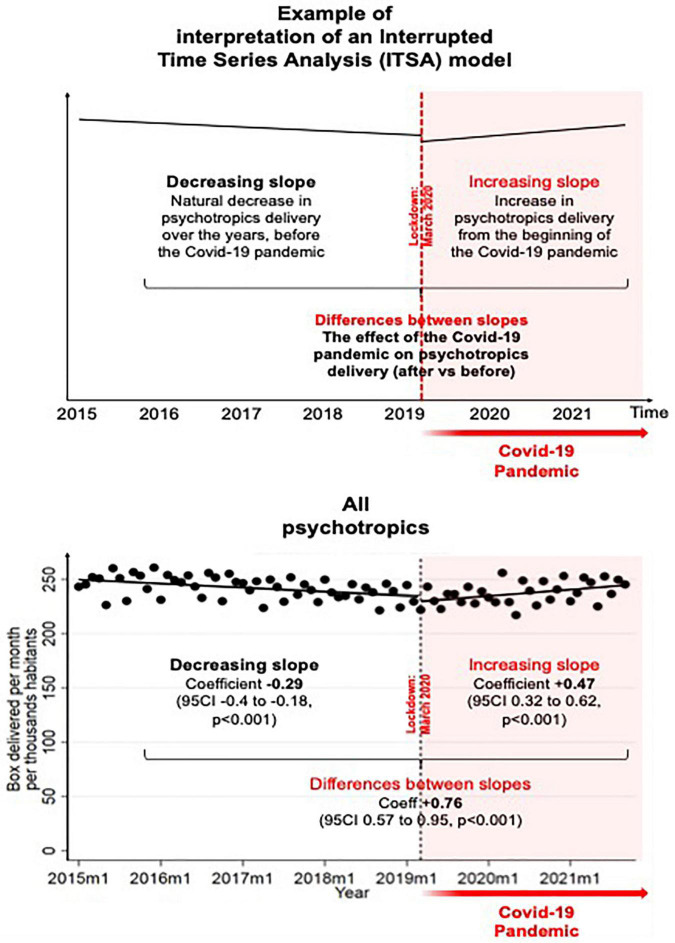
Influence of the COVID-19 pandemic on the psychotropic drugs delivery.

In January 2015, there were around a total of 250 boxes of psychotropic medication delivered per month and per thousand inhabitants, with a progressive decrease to reach around 232 boxes in March 2020. Statistically, the delivery of all psychotropic drugs was decreasing by −0.29 (95 CI −0.40 to −0.18, *p*<0.001) boxes per month per thousand inhabitants between January 2015 and March 2020, whereas there was an increase of 0.47 (0.32 to 0.62, *p*<0.001) boxes per month per thousand inhabitants following the first lockdown in March 2020. The differences between slopes before and after the first lockdown is a relative increase of 0.76 (0.57 to 0.95, *p*<0.001) boxes per month and per thousand inhabitants. In September 2021, the total number of boxes delivered nearly reached the level of 2015 (Figure 2).

### The influence on each class of psychotropic medication

In January 2015, there were around 77 boxes of antidepressants delivered per month per thousand inhabitants, with a slight increase to reach 82 boxes in March 2020. Statistically, the prescription of antidepressants was growing by 0.08 (0.05 to 0.11, *p*<0.001) boxes per month per thousand inhabitants from January 2015 to March 2020, whereas there was an increase of 0.31 (0.23 to 0.39, *p*<0.001) boxes per month per thousand inhabitants from March 2020. The difference between slopes before and after the first lockdown is a relative increase of 0.23 (0.15 to 0.32, *p*<0.001) boxes per month per thousand inhabitants. In September 2021, 88 boxes were delivered per month per thousand inhabitants.

In January 2015, there were around 93 boxes of anxiolytics delivered per month per thousand inhabitants, with a decrease to reach 86 boxes in March 2020. Statistically, there was a decrease of −0.12 (−0.16 to −0.08, *p*<0.001) boxes per month per thousand inhabitants before the pandemic, followed by an increase of 0.15 (0.09 to 0.21, *p*<0.001) boxes after the first lockdown, and therefore a relative increase of 0.27 (0.20 to 0.34, *p*<0.001) boxes per month per thousand inhabitants. In September 2021, the dispensing rate of anxiolytics was 89 boxes per month per thousand inhabitants.

In January 2015, there were around 56 boxes of hypnotics delivered per month per thousand inhabitants, with a decrease to reach 40 boxes in March 2020. Statistically, there was a decrease by −0.27 (−0.31 to −0.22, *p*<0.001) boxes per month per thousand inhabitants before the pandemic, following by a flattening of delivery at −0.03 (−0.070 to 0.005, *p* = 0.086) boxes from the first lockdown. The difference between slopes before and after the first lockdown is a relative increase of 0.23 (0.17 to 0.30, *p*<0.001) boxes per month per thousand inhabitants. In September 2021, the dispensing rate of hypnotics was 39 boxes per month per thousand inhabitants.

**FIGURE 3:**
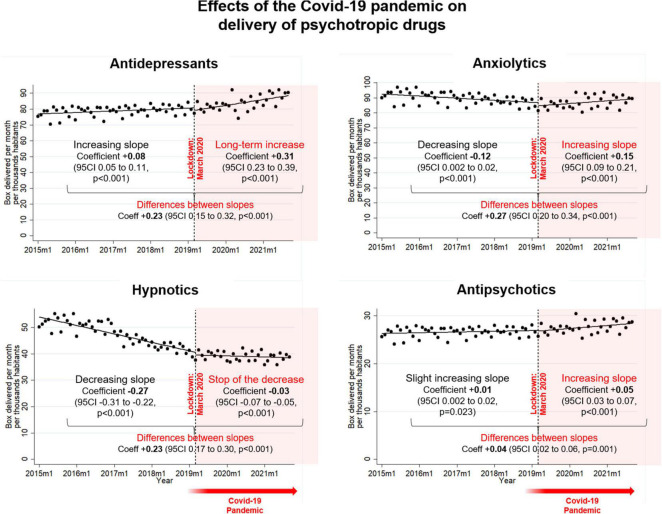
Influence of the COVID-19 pandemic on the psychotropic drugs delivery, by class of psychotropic drugs.

**FIGURE 4:**
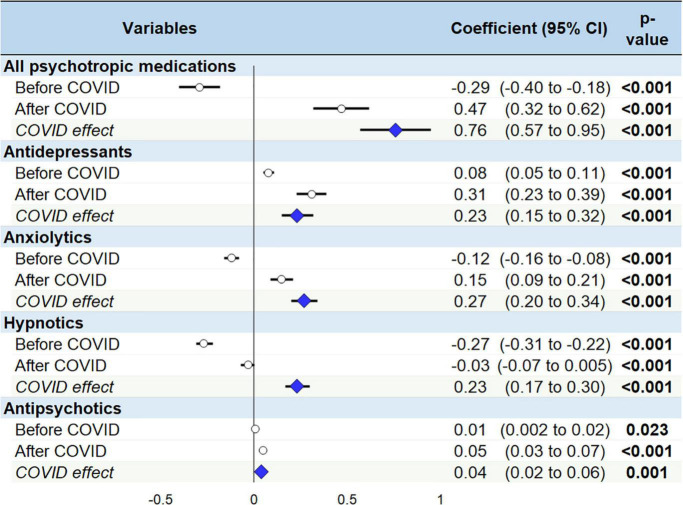
Slopes and the COVID-19 effect on the psychotropic drugs delivery.

In January 2015, there was around 26 boxes of antipsychotics delivered per month per thousand inhabitants, with nearly the same rate in March 2020. Statistically, there was a slight increase of 0.01 (0.002 to 0.020, *p* = 0.023) boxes before the pandemic, followed by an increase of 0.05 (0.03 to 0.07, *p*<0.001) boxes per month per thousand inhabitants after March 2020. The difference between slopes before and after the first lockdown is a relative increase of 0.04 (0.02 to 0.06, *p* = 0.001) boxes per month per thousand inhabitants. In September 2021, the dispensing rate of antipsychotics was 28 boxes per month per thousand inhabitants (Figures 3, 4).

## Discussion

The main findings were characterized by a clear break in the long-term delivery of psychotropic drugs. Antidepressants, anxiolytics, and hypnotics had the strongest change in trend marked by an increase in delivery after the pandemic, relative to the long-term trend before. In the other hand, antipsychotics underwent a very slight and perceptible increase.

### Psychotropic consumption

Our results showed a decrease in the prescription of anxiolytics and hypnotics during the pre-pandemic period, whereas antidepressants were on the rise. The decrease in anxiolytics and hypnotics and the increase in antidepressants could correspond to better compliance with good practice recommendations (23). Many studies have been conducted since the beginning of the pandemic on mental health. These studies showed an upsurge in anxiety, stress, and depressive symptoms, directly linked with the health situation (4–8). Our results corroborate these data at a national level. Moreover, the delivery of psychotropic drugs was objectively measurable and independent of any observer. Despite two studies showing an increase in the delivery of psychotropic drugs during the COVID-19 pandemic, our study had an exhaustive sample size (24) and a long-term follow-up (25). On the opposite, some studies did not find significant variation in the prescription of psychotropic drugs following the pandemic (26–30) that may be explained by specificities of the lockdown in each country. For example, Sweden, (30) Poland, (26) or Northern California (29) showed only an isolated peak in March 2020. The discordance of results between countries may be explained by difference in the severity of lockdown restrictions, with a variable impact on daily life. (31) The French government imposed several measures to limit the spread of the disease such as strict containment, with schools closed and non-essential activities halted (32). Certainly, the first lockdown has left its mark by the violence of the changes it has caused in the daily life of the general population (2). In France, mental health assessment indicators showed an approximatively 13% increase in anxiety when comparing a test period in 2017 and the COVID-19 pandemic. Depression increases by 10%, especially during periods of lockdown. Sleep disturbance, meanwhile, increases by 12% at the beginning of the pandemic to a 14% increase by the end of our study period in September 2021 (33). Our results showed the increase in the delivery of psychotropic drugs, in line with the increase in the prevalence of these symptoms in the French population. Several studies on containment measures implemented during past epidemics showed that these social isolation measures increased anxiety, stress, and depressive symptoms (3). A frequent increase in the delivery of stress-related medication has also been shown in the aftermath of a traumatic or stressful event affecting the general population (14–16). The global lockdown of more than 4 billion people simultaneously, with alarming media coverage centered on the pandemic (daily number of COVID-19 cases and daily deaths), can surely be considered as a traumatic event (34–36). The extensive use of social media further accentuated this psychological distress (37). The novelty of our results also lies in the long-term increase of psychotropic drugs delivery. The months that followed the first lockdown maintained a climate of anxiety with successive waves of contamination and restrictions. During the time of our study, France experienced three strict lockdowns, interspersed with curfew periods, that where somewhat anxiogenic. These long-time measures have undermined the resilience of the population, with a feeling of endless situation and contributed to the development of COVID-19 psychological fatigue (38). The fear of an economic crisis due to the pandemic also contributed to this anxiety (39, 40). This prolonged exposure to a state of high stress led to the development of post-traumatic stress disorder (PTSD) in some patients, health care workers, and even in the general population (41–43).

### Disparities between classes of psychotropic drugs

Our results showed an increase in the delivery of psychotropic drugs since the beginning of the pandemic. Nevertheless, there were some disparities according to the therapeutic class studied. Indeed, the increase in antidepressants, anxiolytics and hypnotics delivery were similar in proportion. Some studies already demonstrated the negative impact of the COVID-19 pandemic on mental health, using self-administrated questionnaires or medical interview-based epidemiological surveys. (31, 42, 44–46) These studies showed the increase in depressive symptoms, anxiety and sleep disorders. (10, 33, 44, 47) Our study confirmed the severity of those symptoms, that were translated into a global increase in the delivery of antidepressants, anxiolytics, and hypnotics. Most effective public health measures to prevent depressive symptoms, anxiety and sleep disorders are behavioral such as practicing a regular physical activity, (48, 49) and having social interaction. Both were particularly limited during the first lockdown (50–52). Therefore, despite the fear of the COVID-19 itself, the lockdown and its associated behavioral restriction might have increase prevent depressive symptoms, anxiety, and sleep disorders. For the most severe patients, some non-medication approach exists to treat depressive symptoms, anxiety and sleep disorders, such as intervention of a mobile psychiatric team, a close follow-up by a psychiatric nurse, or psychotherapy (53, 54). However, the first lockdown was marked by a limitation in the access to psychiatric structures (55, 56). Access to care and medical prescriptions was extremely difficult in this period, as patients had no possibility to consult in outpatient facilities. Only patients requiring hospitalization were treated in psychiatric structures. However, our study does not consider hospital prescriptions. The increase in antidepressants, anxiolytics, and hypnotics may be explain by the prescription from private practice medical doctors, particularly general practitioners who were also in front-line during the first lockdown (57). Then, the rise in antidepressants, anxiolytics, and hypnotics may be explained by the addition of telephone hotlines, that took some time to set up, even if they could not replace structures. Alarmingly, despite the reopening of those structure in the following months, the symptoms were judged sufficiently severe by medical doctors to require a drug treatment. The pandemic may in fact have been composed of several consecutive periods, each of them having some specificities that might have impacted psychotropic drug delivery. Further studies with daily data (and not monthly as in our study), should analyze the different periods of the pandemic separately. Despite we did not have data on symptoms, the analysis of the administrative databases of drug delivery is a complementary and necessary approach to follow mental health of population (11). Our study did not show a significant change in the delivery of antipsychotics. A recent systematic review of case reports and case series of psychosis onset during the COVID-19 pandemic discussed rare cases (58). Therefore, the pandemic did not seem to have massively increased new-onset psychosis. However, even psychotic patients had a significant increase in symptoms of depression and anxiety (59). Moreover, the difficulties of follow-up associated with the health situation made these patients more sensitive than the general population to the deterioration of mental health secondary to the pandemic (60).

### Limitations

Our study may suffer from some limitations. We do not have basic information such as sociodemographic (age, sex), delivery localization, prescribers or details about medication packaging allowing us to compute a defined daily dose (DDD). However, we used a massive database derived from the SNDS, that include more than 99% of the French population. Since all patients are extracted from a national database, there is no study selection bias, nor attrition or generalizability bias. Our database gives us information on the delivery and not on the consumption of psychotropic drugs and does not include hospital dispensing. The French population being a large consumer of psychotropic drugs, (61) several individuals may have taken medications already at home. Despite an increase in the use of these stocks would go unnoticed in our study, our results provide a high level of evidence for an increase of delivery of psychotropic drugs because of the pandemic. We chose to use an international classification to gather drugs, which may have hidden variations specific to certain drugs. Moreover, the data at our disposal were aggregated by months, therefore preventing to study precisely particular periods such as the effects of the three lockdowns on treatment delivery. Further studies should compare national databases from different countries, in the light of specific restrictions measures.

## Conclusion

The COVID-19 pandemic resulted in a significant increase in the dispensing of psychotropic drugs to the general population in France. The increase was most noticeable for antidepressants, anxiolytics, and hypnotics. Further studies are needed to explain the increase in psychotropic drug dispensing during the pandemic, as well as the population most at risk, in order to build effective preventive strategies.

## Data availability statement

Publicly available datasets were analyzed in this study. This data can be found here: https://assurance-maladie.ameli.fr/etudes-et-donnees/medicaments-classe-atc-medicam-2021.

## Author contributions

FD: conceptualization, methodology, formal analysis, visualization, and supervision. PB: investigation and writing—original draft preparation. AM: data curation. PV, GB, J-BB-M, VF, AM, and FD: writing—review and editing. All authors have read and agreed to the published version of the manuscript.
